# Administration of JTE013 abrogates experimental asthma by regulating proinflammatory cytokine production from bronchial epithelial cells

**DOI:** 10.1186/s12931-016-0465-x

**Published:** 2016-11-09

**Authors:** Tomomi Terashita, Kazuyuki Kobayashi, Tatsuya Nagano, Yoshitaka Kawa, Daisuke Tamura, Kyosuke Nakata, Masatsugu Yamamoto, Motoko Tachihara, Hiroshi Kamiryo, Yoshihiro Nishimura

**Affiliations:** Division of Respiratory Medicine, Department of Internal Medicine, Kobe University Graduate School of Medicine, 7-5-1 Kusunoki-cho, Chuo-ku, Kobe, 650-0017 Japan

**Keywords:** Sphingosine-1-phosphate, epithelial cells, BEAS-2B, JTE013, CCL3

## Abstract

**Background:**

Sphingosine-1-phosphate (S1P) is a bioactive phospholipid that acts as a signal transducer by binding to S1P receptors (S1PR) 1 to 5. The S1P/S1PRs pathway has been associated with remodeling and allergic inflammation in asthma, but the expression pattern of S1PR and its effects on non-immune cells have not been completely clarified. The aim of this study was to examine the contribution of the signaling of S1P and S1PRs expressed in airway epithelial cells (ECs) to asthma responses in mice.

**Methods:**

Bronchial asthma was experimentally induced in BALB/c mice by ovalbumin (OVA) sensitization followed by an OVA inhalation challenge. The effects of S1PR antagonists on the development of asthma were analyzed 24 h after the OVA challenge.

**Results:**

Immunohistological analysis revealed S1PR1-3 expression on mouse airway ECs. Quantitative real-time polymerase chain reaction demonstrated that S1P greatly stimulated the induction of *CCL3* and *TIMP2* mRNA in human airway ECs, i.e., BEAS-2B cells, in a dose-dependent manner. Pretreatment with the S1PR2 antagonist JTE013 inhibited the *CCL3* gene expression in BEAS-2B cells. Immunohistological analysis also showed that the expression level of *CCL3* was attenuated by JTE013 in asthmatic mice. Furthermore, JTE013 as well as anti-CCL3 antibody attenuated allergic responses. Intratracheal administration of JTE013 also attenuated eosinophilic reactions in bronchoalveolar lavage fluids. S1P induced transcription factor NFκB activation, while JTE013 greatly reduced the NFκB activation.

**Conclusions:**

JTE013 attenuated allergic airway reactions by regulating CCL3 production from bronchial ECs. The intratracheal administration of JTE013 may be a promising therapeutic strategy for bronchial asthma.

**Electronic supplementary material:**

The online version of this article (doi:10.1186/s12931-016-0465-x) contains supplementary material, which is available to authorized users.

## Background

Sphingosine-1-phosphate (S1P) is a bioactive phospholipid metabolite [1]. It is produced by most kinds of cells through phosphorylation by sphingosine kinases (SPHK1 and SPHK2), and it works as a signal transducer of intracellular and extracellular cell homeostasis and functions, such as cell differentiation, inflammation, and apoptosis [2]. Outside of the cell, S1P binds to five G-protein-coupled receptors, i.e., S1P receptors (S1PR) 1 to 5, as a ligand. These receptors are expressed mainly by inflammatory cells, and they play an important role in immunity. The individual expression pattern of S1PRs on each cell creates diverse immunological responses [3]. However, the expression pattern of S1PR and its effects on non-immune cells remain unclear. Therefore, we performed a functional analysis of sphingolipids focusing on the roles of S1P and S1PRs on non-immune cells. First, we reported that TGF-β increases SPHK1, and consequently S1P, which transactivates S1PR2 and S1PR3 on fibroblasts, and promotes their transformation to myofibroblasts through Rho kinase [4, 5]. We further reported that SPHK1 mediates the transformation of airway epithelial cells (ECs) to goblet cells [6]. We also clarified that the intratracheal administration of N,N-dimethylsphingosine (DMS), a S1P synthesis inhibitor, restrained allergic inflammation and remodeling [7]. Thus, S1P and S1PRs play crucial roles in remodeling and allergic inflammation in asthma.

Bronchial asthma is a cytokine-based inflammatory dysfunction that involves airway ECs, endothelial cells, and inflammatory cells, such as Th2-type lymphocytes, dendritic cells, and mast cells [8]. Healthy airway ECs, which provide a physical barrier, can keep mast cells in a quiescent state and inhibit mast cell degranulation [3]. However, damage to airway ECs due to inflammation initiates innate immune responses, and stimulates Th2 responses by directly attracting and activating immune cells, such as Th2 cells, eosinophils, and mast cells [9–13]. Clarification of the functions of airway ECs and efforts to minimize airway EC damage are important for asthma treatment.

In this study, we investigated the expression of S1PRs on airway ECs and the function of airway ECs in the signaling pathway of the S1P/S1PRs axis by using an experimental bronchial asthma mouse model. We used human bronchial ECs, i.e., BEAS-2B cells, stimulated with S1P for identifying related cytokines to investigate the signaling pathway, and to evaluate the potential of S1PR inhibitors as a remedy for bronchial asthma.

## Methods

### Reagents

S1P (PeproTech, Rocky Hill, NJ) was used. A S1PR2 antagonist (also known as JTE013; Cayman, Ann Arbor, MI), and a S1PR3 antagonist (also known as VPC23019; Calbiochem, Darmstadt, Germany) were purchased. The antibodies used were anti-S1PR1-5 (Cayman) and anti-CC chemokine ligand (CCL) 3 (R&D Systems, Minneapolis, MN).

### Animals

Female BALB/c mice were purchased from SLC Japan (Shizuoka, Japan). All mice were raised in sterile cages, and were used at 7 to 10 weeks of age. Our studies were approved by the Institutional Animal Care and Use Committee (Permit Numbers: P130610-R1, and P150804), and carried out in accordance with the Kobe University Animal Experimentation Regulations.

### Experimental mouse models

The experimental bronchial asthma model mice were prepared by sensitization with intraperitoneal (i.p.) injections of 10 μg of ovalbumin (OVA; Sigma-Aldrich, St. Louis, MO) and 2 mg of aluminum hydroxide (Sigma-Aldrich) in 0.5 ml of sterile phosphate-buffered saline (PBS) on days 0 and 7. Then, on days 21 and 22, the mice were exposed to aerosolized sterile PBS with or without 1.0 % OVA for 30 min with ultrasonic nebulizer, Omron NE-U07 (OMRON, Kyoto, Japan) (the aerosolized particle size ranges 1–8 μm). Mice were sacrificed on day 23 for all mice experimentations. To prepare JTE013-treated or anti-CCL3 antibody-treated mice, mice were given JTE013 (4 mg/kg) or anti-CCL3 antibody (0.15 mg/kg) via i.p. injection before OVA sensitization on days 21 and 22. In the JTE013 intratracheal administration experiment, mice were intratracheally instilled with 10 μg of JTE013 or PBS.

### Analysis of bronchoalveolar lavage fluids

Bronchoalveolar lavage (BAL) fluids were collected by lavaging lungs three times with 800 μl of PBS, and samples were stored at −80 °C after centrifugation. The concentrations of interleukin (IL)-4, IL-5, IL-13, and IFN-γ in the BAL fluids were measured using the Mouse ELISA Kit (R&D Systems) according to the manufacturer’s instructions.

### Histology

Lungs were harvested to prepare paraffin-embedded sections for histological analysis. PBS was perfused through the lungs of mice under deep anesthesia, and the lungs were then fixed by intratracheal instillation of phosphate-buffered paraformaldehyde (Wako, Osaka, Japan). Airway EC areas were evaluated in five randomly selected sections, and each section was subjected to hematoxylin and eosin staining and immunostaining, as previously described [14, 15].

### Cell culture and stimulation

BEAS-2B cells (ATCC, Manassas, VA) were incubated with the BEGM Bullet Kit (Lonza, Walkersville, MD) under a humidified atmosphere containing 5 % CO_2_ at 37 °C. To analyze the secretion of cytokines, the cells were stimulated with or without S1P (100 nM or 1 μM) for 3 h. In other experiments, cells were treated with 1 μM S1P and 10 μM JTE013, 10 μM VPC23019, 50 μM S3I-201 (STAT3 inhibitor, Cosmo Bio Co, Tokyo, Japan), 2.9 μM IKK inhibitor III (Calbiochem), or dimethyl sulfoxide (DMSO).

### Quantitative real-time polymerase chain reaction analysis

Total cellular RNA was prepared and quantitative real-time polymerase chain reaction (qRT-PCR) was performed as previously described [16]. Relative human mRNA levels were calculated with the ΔΔCt method using glyceraldehyde-3-phosphate dehydrogenase mRNA as an internal control. The sequences of the sense and antisense primers are listed in Additional file 1: Table S1.

### Western blotting analysis

The preparation of cell lysates, sodium dodecyl sulfate-polyacrylamide gel electrophoresis, and immunoblotting were performed as previously described [17]. Primary antibodies against NFκB (Santa Cruz Biotechnology, Dallas, TX) and β-actin (Cell Signaling, Danvers, MA), and secondary antibodies (Cell Signaling) were used.

### Statistical analysis

Data are shown as the mean ± standard error of the mean (SEM). Statistical analyses were performed using the Student’s unpaired *t*-test to compare two groups. The significances of all statistical tests are provided in the figures. A *p*-value of <0.05 was considered to be statistically significant.

## Results

### S1PR1-3 is expressed on airway epithelial cells in vivo

**Fig. 1:**
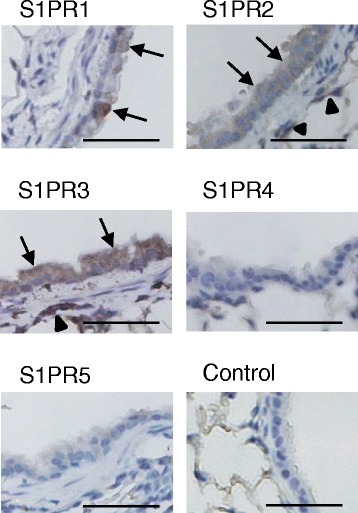
S1PR1-3 is expressed on airway epithelial cells in vivo. Lung sections from BALB/c mice immunostained with antibodies against S1PR1-5 and without these antibodies (control IgG). Bronchial ECs are stained with S1PR1-3 antibodies (*arrows*), and alveolar ECs are stained with S1PR2 and 3 antibodies (*arrowheaads*)

Previous studies suggested the existence of S1PRs on airway ECs, because the administration of DMS to ECs mitigated allergic reactions [6, 7]. Thus, we investigated the localization of S1PRs on ECs by immunostaining with S1PRs antibodies. As shown in Fig. 1, S1PR1, S1PR2, and S1PR3 were expressed in airway ECs, including bronchial ECs and alveolar ECs. These results suggest that the S1P/S1PR1-3 axis in airway ECs has a possible role in airway inflammation.

### S1P stimulation of airway ECs evidently induces *CCL3* and *TIMP2* gene expression in vitro

**Fig. 2:**
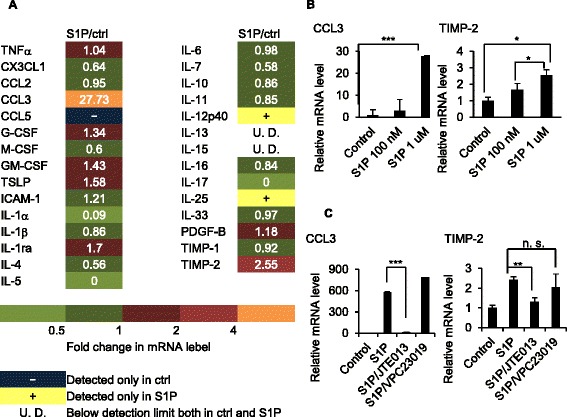
S1P stimulation of airway ECs induces *CCL3* and *TIMP2* gene expression, and* CCL3 *and *TIMP2* are S1P-dependent in vitro. BEAS-2B cells were cultured with or without S1P (100 nM). The mRNA expression of 29 cytokines was analyzed by quantitative real-time RT-PCR. Data represent the ratio between the relative mRNA level of S1P-treated cells and that of S1P-untreated cells (**a**). BEAS-2B cells were treated with S1P (100 nM or 1 μM) (**b**), S1P (1 μM) and JTE013 (10 μM), or S1P (1 μM) and VPC23019 (10 μM) (**c**) for 3 h, and CCL3 and TIMP2 gene expression was analyzed by quantitative real-time RT-PCR

Next, we analyzed the function of the S1P/S1PR1-3 axis in cytokine secretion by using BEAS-2B human airway ECs. Comparison by qRT-PCR of the cytokine mRNA levels of S1P- or DMSO-treated BEAS-2B cells indicated that stimulation with S1P promoted the expression of *CCL3* and *TIMP2* (Fig. 2a).

### *CCL3* and *TIMP2* are S1P-dependent in vitro and in vivo

**Fig. 3:**
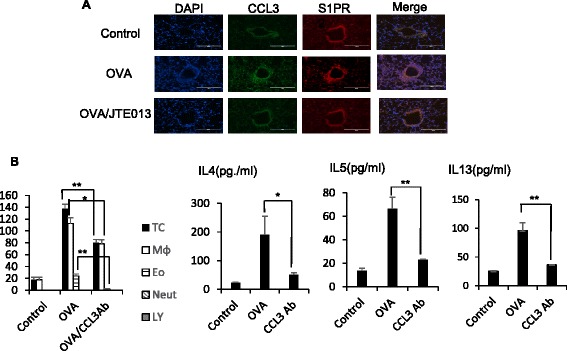
*CCL3* and *TIMP2* are S1P-dependent in vivo. Immunofluorescent microscopic images show OVA-treated lung sections stained with FITC-conjugated anti-CCL3 (*green*), and Alexa 594-conjugated anti-S1PR2 (*red*) antibodies. Co-localization of CCL3 and S1PR2 is seen in *yellow* (**a**). BAL Fluids were obtained from BALB/c mice treated with vehicle, OVA, or OVA/mCCL3 antibody (3 μg/cavity), and examined by Diff-Quick staining. The total cell counts and cell differentials in the BAL fluids are shown, and the concentrations of IL-4, IL-5, and IL-13 in the BAL fluids were measured by using ELISA kits (**b**). Data are expressed as the mean ± standard error (SE) of at least three independent experiments. *p < 0.05, **p < 0.01, ***p < 0.001

We further analyzed the dose-dependent *CCL3* and *TIMP2* gene expression in BEAS-2B cells after stimulation with S1P. As shown in Fig. 2b, *CCL3* and *TIMP2* gene expression in BEAS-2B cells increased in proportion to the S1P concentration, and they were attenuated by JTE013, a S1PR2 antagonist (Fig. 2c). In contrast, neither was attenuated by VPC23019, a S1PR1 and S1PR3 antagonist (Fig. 2c). Immunohistological analysis also showed that CCL3 and S1PR2 were co-expressed on the airway ECs in the experimental asthma mouse model, and the expression level of CCL3 was attenuated by JTE013, although the expression level of S1PR2 was not attenuated by JTE013 because JTE013 only inhibits S1P binding to S1PR2 (Fig. 3a). further analyzed the effect of CCL3 on airway allergic response using the experimental asthma mouse model. As shown in Fig. 3b, airway eosinophilia and the levels of IL-4, IL-5, and IL-13 were attenuated by the anti-CCL3 antibody. These results suggest that S1P induced the secretion of CCL3, which has a crucial role in bronchial asthma through the S1P/S1PR2 axis in airway ECs.

### JTE013 decreases allergic responses

**Fig. 4:**
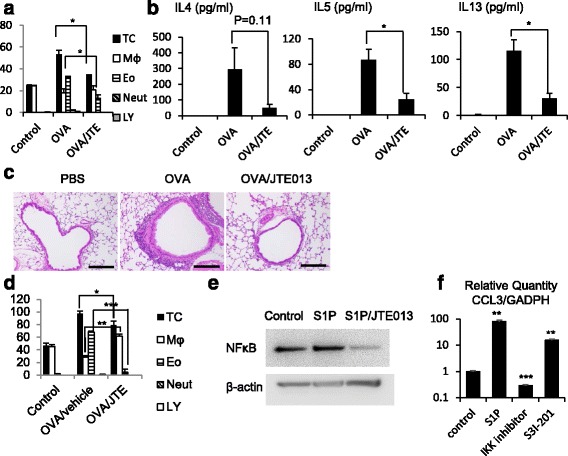
JTE013 decreases allergic responses. BAL fluids were obtained after OVA or vehicle exposure, and examined by Diff-Quick staining. Total cell counts and cell differentials in the BAL fluids are shown (**a**), and the concentrations of IL-4, IL-5, IL-13, and IFN-γ in the BAL fluids were measured by using ELISA kits (*n* = 5) (**b**). Lung sections from BALB/c mice treated with vehicle, OVA, or OVA/JTE013 (4 mg/kg) were subjected to hematoxylin and eosin staining (**c**). BAL fluids were obtained after OVA or vehicle exposure with intratracheal administration of vehicle or JTE013 (10 μg/trachea), and the total cell counts and cell differentials in the BAL fluids were examined by Diff-Quick staining (*n* = 3 to 5) (**d**). BEAS-2B cells were treated with vehicle, S1P (1 μM), or S1P/JTE013 (1 μM) for 4 h. Phosphorylation of p65 was evaluated by western blot analysis. Representative images from three independent experiments are shown (**e**). BEAS-2B cells were treated with vehicle, S1P (1 μM), IKK inhibitor, or S3I-201. Each *CCL3* gene expression was analyzed by quantitative real-time RT-PCR, representing the relative mRNA level of CCL3 (**f**). Data are expressed as the mean ± SE of at least three independent experiments. *p < 0.05, **p < 0.01, ***p < 0.001

To investigate the effects of the S1P/S1PR2 axis on bronchial asthma, we employed an experimental asthma mouse model and evaluated the therapeutic effects of JTE013. As expected, OVA-treated mice showed a marked increase in the number of eosinophils in the BAL fluids (Fig. 4a). In addition, the levels of IL-4, IL-5, and IL-13 were increased in the BAL fluids from OVA-treated mice (Fig. 4b). In contrast, JTE013 administration resulted in a significant decrease in Th2 inflammatory reactions, although there was no statistical difference in the levels of IL-4 (Fig. 4a and b). Consistent with the BAL fluids analysis, histological analysis demonstrated a marked decrease in inflammatory cell infiltration around the bronchiole in OVA/JTE013-treated mice when compared to OVA-treated mice (Fig. 4c).

Moreover, intratracheal administration of JTE013 also resulted in a marked decrease in eosinophilic reactions in the BAL fluids (Fig. 4d). This suggests that intratracheal treatment can inhibit allergic bronchial inflammation as well as i.p. treatment.

### JTE013 inhibits *CCL3* gene expression by attenuating NFκB and STAT3 activation in vitro

Next, we analyzed the signaling pathways downstream of S1PR2, and investigated the activation of transcription factors, NFκB and STAT3. Previous study reported that S1PR2 can activate the transcription factor, STAT3 in mice lung [18], which regulates CCL3 expression in macrophage [19], and the transcription factor NFκB also induces CCL3 synthesis in nucleus pulposus cells [20]. In this study, using BEAS-2B cells, we first assessed the activity of NFκB downstream of the S1P/S1PR2 signaling pathway, and next analyzed the CCL3 expression downstream of NFκB and STAT3 activation. The results are shown in Fig. 4e and f. The expression of NFκB increased after stimulation with S1P, while it decreased with JTE013 (Fig. 4e), and *CCL3* gene expression in BEAS-2B cells increased with the S1P concentration, while it was attenuated by IKK inhibitor, and a STAT3 inhibitor, S3I-201. Taken together, these results suggest that JTE013 inhibits CCL3 expression through NFκB and STAT3 transcription.

## Discussion

The aim of this study was to elucidate the role of S1P in bronchial asthma by focusing on airway ECs. Pathological analysis showed that S1PR1-3 are expressed on airway ECs, and airway ECs that expressed S1PR2 also expressed CCL3 at a high level in response to the OVA inhalation challenge. Our previous research showed the inflammation induced by OVA exposure decreases in time-dependent manner [17]. Therefore, we adopted our time schedule as appropriate duration time.

In vitro analysis of cytokine expression in human bronchial ECs also showed that S1P led to the transcriptional activation of the *CCL3* gene through NFκB and STAT3 activation. Previous studies reported that S1PR2 is coupled to GαI, Gα12/13, and GαQ, which activates phospholipase C (PLC) [21, 22]. PLC plays a role in the regulation of intracellular signaling pathways through calcium release and protein kinase C activation [22]. PLC also plays a role in proinflammatory gene expression through the activation of NFκB [23]. These results suggest that the S1P/S1PR2 axis has a possible role in cytokine expression via the activation of NFκB downstream of PLC. Furthermore, STAT3 is directly activated by S1PR2 in a Rho-dependent manner [18, 24], which is dependent on the S1PR2-coupled G protein Gα12/13 [21].

We used human bronchial ECs instead of mouse bronchial ECs as previous literatures [25–27], because previous reports showed that approximately 99 % of mouse genes have a homologue in the human genome [28], and bioinformatic analysis and human curation of 60,770 full-length mouse cDNA clones showed similarity (70–85 % identity) to known human-disease genes [29].

**Fig. 5:**
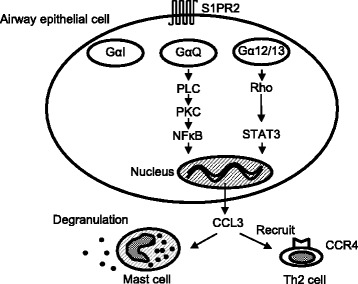
Simplified scheme of the S1PR2 signaling events in airway epithelial cells. On airway epithelial cells, S1P binds to S1PR2, which is coupled to GaI, Ga12/13, and GaQ. GaQ in turn activates PLC, activating PKC. PKC phosphorylates NFκB, which binds to the proximal promotor of CCL3 major nuclear proteins. In addition, another S1PR2-coupled G protein, Ga12/13, also up-regulates CCL3 expression through STAT3 activation in a Rho-dependent manner

CCL3 expression is up-regulated by NFκB and STAT3. Consistent with our results, Jianru W et al. also demonstrated that NFκB introduces CCL3 synthesis, by binding to the proximal promotor of CCL3 major nuclear proteins in nucleus pulposus cells [20]. Furthermore, Zhang C et al. also reported that STAT3 is a key regulator of CCL3 expression in macrophages [19]. Taken together, we infer that the S1P/S1PR2 axis activates STAT3 and NFκB to form a complex with CCL3 nuclear protein binding sites, which in turn produces CCL3 through Gα12/13 and GαQ (Fig. 5).

CCL3 is a member of the C-C chemokine family, and is a chemoattractant for eosinophils, lymphocytes, leukocytes, and monocytes. Previous studies reported that an increased level of CCL3 was observed in BAL fluids from patients with asthma [30], and that CCL3 evoked local T cell recruitment and induced skin reactions in patients with atopic dermatitis [31]. Furthermore, Elena et al. demonstrated significantly higher levels of CCL3 in BAL fluids from patients with corticosteroid-resistant asthma [32].

CCL3 is also a ligand for CCR1 and CCR4. CCR1 is expressed in mast cells and basophils, and the CCL3/CCR1 axis is important for the activation of mast cells [33]. A previous study reported that CCL3 acted as a co-stimulator for FcεRI-mediated degranulation in conjunctival mast cells in CCL3-deficient mice [34]. Moreover, CCL3 synergistically enhanced FcεRI-mediated degranulation and the production of CCL2, which is a chemoattractant for T cells and eosinophils in bone marrow-derived murine mast cells and the rat basophilic leukemia 2H3 cell line [33, 35–39]. Taken together, there is crosstalk between CCR1-mediated and FcεRI-mediated signaling cascades in mast cells, which plays an important role in allergic disorders as FcεRI cross-linking is a key event of activation. On the other hand, CCR4 mediates allergy-promoting Th2 cell recruitment [23]. A recent publication by Oskeritzian et al. demonstrated that S1P neutralization or a S1PR2 antagonist strongly attenuated early T-cell recruitment to lung perivascular lesions and T-cell chemokine production, including CCL3, in murine mast cell-dependent acute airway allergic responses [23], suggesting that the S1P/S1PR2 axis regulates early T-cell recruitment via chemokine production. In our study, immunohistochemical analysis showed that CCL3 was abundantly produced by airway ECs. Therefore, CCL3 on airway ECs may be a promising target for the treatment of airway allergic inflammation, which is mediated by T-cell recruitment. In fact, our study showed that CCL3 neutralization actually reduced Th2-type allergic responses.

The importance of S1P in an experimental asthma mouse model is well established [4–7]. Past reports have suggested that the S1P/S1PR2 axis also plays an important role in antigen-induced mast cell degranulation [40], myofibroblast contraction [41], and vasoconstriction [42]. Our study first verified the contribution of the signaling of the S1P/S1PR2 axis in airway ECs. However, the intraperitoneal administration of S1PR2 antagonist to investigate the in vivo reaction of airway ECs can also induce a systemic reaction, as described above. Although we examined the intratracheal administration of a S1PR2 antagonist, the possibility cannot be excluded that the method could also induce systemic reaction through resident mast cells around airway ECs. In order to determine the effects of the S1PR2 antagonist on airway ECs in vivo, an appropriate experimental mouse model, such as S1PR2-deficient mice [43], should be employed.

Our in vitro study using primary cultures demonstrated that the S1P/S1PR2 axis promotes airway inflammation. Intriguingly, Yapeng et al. showed that S1PR2 induced the extrusion of apoptotic kidney ECs to maintain an intact barrier [44]. In addition, Kono et al. showed that S1PR2-knockout mice had defective epithelial barrier function in the cochlea [45]. These results suggest that inhibition of the S1P/S1PR2 pathway disturbs the barrier function of ECs, and may aggravate asthmatic reactions. We have two hypotheses to account for these contradictory findings. One hypothesis is that the nature of airway ECs is different from those of other ECs. The other hypothesis is that the quantity of JTE013 used was enough to restrain inflammatory responses, but not enough to restrain the extrusion of airway ECs. Further studies investigating the effects of the S1PR2 antagonist at different intratracheal dosage levels on asthma would be of great interest.

In addition, we investigated the allergic reaction by intratrachial administration of aerosolized OVA and JTE013 to show the potential of intratrachial administration of JTE013 as the remedy of bronchial asthma. However, Kannan et al. have revealed that the deposition of particle substance to airway and lung depends on many factors including the property of the particle, anatomical geometry of the airway, and respiratory pattern [46, 47]. And it may be possible to obtain more analysis from the computational Euler-Langrangian particle delivery approach of Kannan et al. [47]. In this point, there are some limitations to interpret our in vivo data. Though extrapolating from our studies with intratrachial administration in mouse model to the allergic reaction in humans requires cautious interpretation, our data can provide significant promise to JTE013 as the remedy because previous study showed that asthma mouse model can be used to investigate the main point of our pathogenesis, allergic reaction through the cytokine production [48] and previous report showed the intratrachial administration of drugs such as budesonide in mice has similar efficacy in humans [49]. Therefore, I think our results can lead to the development of worthwhile therapeutic interventions.

## Conclusion

This study demonstrated that the S1PR2 antagonist JTE013 inhibits asthmatic allergic reactions by suppressing the S1PR2-mediated NFκB activation and CCL3 production in the bronchial epthelium, and that the intratracheal administration of JTE013 may be a promising remedy for bronchial asthma.

## Additional file

Additional file 1: Table S1. The primers used for qRT-PCR. (DOCX 30 kb)

## Abbreviations

BAL: Bronchoalveolar lavage
CCL: CC chemokine ligand
DMS: N,N-dimethylsphingosine
DMSO: Dimethyl sulfoxide
ECs: Epithelial cells
i.p.: Intraperitoneal
IL: Interleukin
OVA: Ovalbumin
PBS: Phosphate buffered saline
PLC: Phospholipase C
qRT-PCR: Quantitative real-time polymerase chain reaction
S1P: Sphingosine-1-phosphate
S1PR: Sphingosine-1-phosphate receptor
SEM: Standard error of the mean
SPHK: Sphingosine kinase
