# Supplementary material for: Administration of JTE013 abrogates experimental asthma by regulating proinflammatory cytokine production from bronchial epithelial cells
Source: Respir Res. 2016 Nov 9;17:146. doi: 10.1186/s12931-016-0465-x (PMC5103479; doi:10.1186/s12931-016-0465-x)
Supplement: Additional file 1: Table S1. — The primers used for qRT-PCR. (DOCX 30 kb) [file 12931_2016_465_MOESM1_ESM.docx]

**Additional file 1: Table S1. The primers used for qRT-PCR**

| **Gene** | **Forward Primer (5'-3')** | **Reverse Primer (5'-3')** | **References** |
| --- | --- | --- | --- |
| GAPDH | gcaccgtcaaggctgagaac | atggtggtgaagacgccagt | E1 |
| TNFα | agagggaagagttccccagggac | tgagtcggtcacccttctccag | E2 |
| CX3CL1 | accacggtgtgacgaaatgcaacat | cggcaggggtggtcctgggcttcac | E3 |
| CCL2 | cagccagatgcaatcaatgcc | tggaatcctgaacccacttct | E4 |
| CCL3 | caggtctccactgctgcc | cactcagctccaggtcact | E5 |
| CCL5 | ccatgaaggtctccgcggcac | cctagctcatctccaaagag | E6 |
| G-CSF | gagtgtgccacctacaagctgtgcc | cctgggtgggctgcagggcaggggc | E2 |
| M-CSF | ctgaagagctgcttcaccaa | atggtgctgtccttgacaac | E7 |
| GM-CSF | gcatctctgcacccgcccgctcgcc | cctgcttgtacagctccaggcgggt | E2 |
| TSLP | tatgagtgggaccaaaagtaccg | cctcttgtacagctccaggcgggt | E8 |
| ICAM-1 | ggcctcagtcagtgtga | aaccccattcagcgtca | E9 |
| IL-1α | catcgccaatgactcagaggaag | tgccaagcacacccagtagtcttgctt | E2 |
| IL-1β | tggccctaaacagatgaagtgc | gtagtggtggtcggagattcg | E10 |
| IL-1ra | aacagaaagcaggacaagcg | ccttcgtcaggcatattggt | E11 |
| IL-4 | ggcaactttgtccacggacacaag | ggttcctgtcgagccgtttcagga | E12 |
| IL-5 | cgaactctgctgatagccaatg | ccactcggtgttcattacaccaag | E13 |
| IL-6 | ttcaatgaggagacttgcctg | acaacaacaatctgaggtgcc | E14 |
| IL-7 | tgaaggtaaagatggcaaacaa | caatttctttcatgctgtccaa | E15 |
| IL-10 | ccagacccagacatcaaggc | ggccttgctcttgttttcac | E16 |
| IL-11 | tctctcctggcggacacg | aatccaggttgtggtcccc | E17 |
| IL-12p40 | gcagcttcttcatcagggac | agggtactcccagctgacct | E16 |
| IL-13 | tgaggagctggtcaacatca | caggttgatgctccataccat | E18 |
| IL-15 | gaaaccacatttgagaagtatttc | ccattagaagacaaactgttg | E19 |
| IL-16 | ggcctcacacggtttgaag | caatcgtgacaggtccatcag | E20 |
| IL-17 | agagatatccctctgtgatc | taccccaaagttatctcagg | E21 |
| IL-25 | cggaggagtggctgaagtggag | atgggtaccttcctcgccatg | E22 |
| IL-33 | catctggtactcgctgcctgtc | caacaccgtcacctgattcatt | E23 |
| PDGF-B | tcccgaggagctttatgaga | actgcacgttgcggttgt | E24 |
| TIMP-1 | cttctgcaattccgacctcgt | ccctaaggcttggaacccttt | E5 |
| TIMP-2 | ccacccagaagaagagcctgaa | tggacccatgggatgactgtt | E25 |

GAPDH, glyceraldehyde 3-phosphate dehydrogenase; TNFα, tumor necrosis factor α; CX3CL1, Chemokine (C-X3-C motif) ligand 1; CCL, Chemokine (C-C motif) ligand; G-CSF, granulocyte colony-stimulating factor; M-CSF, macrophage colony-stimulating factor; GM-CSF, granulocyte-macrophage colony-stimulating factor; TSLP, Thymic Stromal Lymphopoietin; ICAM-1, Intercellular Adhesion Molecule 1; IL, interleukin; PDGF, platelet-derived growth factor; TIMP, tissue inhibitors of metalloproteinase.

**References**

E1. Hatakeyama Y, Kobayashi K, Nagano T, Tamura D, Yamamoto M, Tachihara M, Kotani Y, Nishimura Y. Synergistic effects of pemetrexed and amrubicin in non-small cell lung cancer cell lines: Potential for combination therapy. Cancer Lett 2014;343:74-79.

E2. de Waal Malefyt R, Abrams J, Bennett B, Figdor CG, de Vries JE. Interleukin 10(IL-10) inhibits cytokine synthesis by human monocytes: an autoregulatory role of IL-10 produced by monocytes. J Exp Med. 1991;174:1209-1220.

E3. Ollivier V, Faure S, Tarantino N, Chollet-Martin S, Deterre P, Combadière C, de Prost D. Fractalkine/CX3CL1 production by human aortic smooth muscle cells impairs monocyte procoagulant and inflammatory responses. Cytokine. 2003;21:303-11.

E4. Greenblatt MB, Sargent JL, Farina G, Tsang K, Lafyatis R, Glimcher LH, Whitfield ML, Aliprantis AO. Interspecies comparison of human and murine scleroderma reveals IL-13 and CCL2 as disease subset-specific targets. Am J Pathol. 2012;180:1080-94.

E5. Lu P, Nakamoto Y, Nemoto-Sasaki Y, Fujii C, Wang H, Hashii M, Ohmoto Y, Kaneko S, Kobayashi K, Mukaida N. Potential interaction between CCR1 and its ligand, CCL3, induced by endogenously produced interleukin-1 in human hepatomas. Am J Pathol. 2003;162:1249-1258.

E6. Pharoah DS, Varsani H, Tatham RW, Newton KR, de Jager W, Prakken BJ, Klein N, Wedderburn LR. Expression of the inflammatory chemokines CCL5, CCL3 and CXCL10 in juvenile idiopathic arthritis, and demonstration of CCL5 production by an atypical subset of CD8+ T cells. Arthritis Res Ther. 2006;8:R50.

E7. Seta N, Okazaki Y, Kuwana M. Human circulating monocytes can express receptor activator of nuclear factor-kappaB ligand and differentiate into functional osteoclasts without exogenous stimulation. Immunol Cell Biol. 2008;86:453-459.

E8. Zhang, K., Shan, L., Rahman, M.S., Unruh, H., Halayko, A.J., Gounni, A.S. Constitutive and inducible thymic stromal lymphopoietin expression in human airway smooth muscle cells: role in chronic obstructive pulmonary disease. Am J Physiol Lung Cell Mol Physiol. 2007;293:L375–L382.

E9. Glushakova O, Kosugi T, Roncal C, Mu W, Heinig M, Cirillo P, Sánchez-Lozada LG, Johnson RJ, Nakagawa T. Fructose induces the inflammatory molecule ICAM-1 in endothelial cells. J Am Soc Nephrol. 2008;19:1712-1720.

E10. Wood IS, Wang B, Trayhurn P. IL-33, a recently identified interleukin-1 gene family member, is expressed in human adipocytes. Biochem Biophys Res Commun. 2009;384:105-109.

E11. Bellehumeur C, Blanchet J, Fontaine JY, Bourcier N, Akoum A. Interleukin 1 regulates its own receptors in human endometrial cells via distinct mechanisms. Hum Reprod. 2009;24:2193-2204.

E12. Atamas SP, Choi J, Yurovsky VV, White B. An alternative splice variant of human IL-4, IL-4 delta 2, inhibits IL-4-stimulated T cell proliferation. J Immunol. 1996;156:435-441.

E12. Lorentz A, Schwengberg S, Mierke C, Manns MP, Bischoff SC. Human intestinal mast cells produce IL-5 in vitro upon IgE receptor cross-linking and in vivo in the course of intestinal inflammatory disease. Eur J Immunol. 1999;29:1496-503.

E14. Faruqi TR, Gomez D, Bustelo XR, Bar-Sagi D, Reich NC. Rac1 mediates STAT3 activation by autocrine IL-6. Proc Natl Acad Sci U S A. 2001;98:9014-9019.

E15. Roato I, Brunetti G, Gorassini E, Grano M, Colucci S, Bonello L, Buffoni L, Manfredi R, Ruffini E, Ottaviani D, Ciuffreda L, Mussa A, Ferracini R. IL-7 up-regulates TNF-alpha-dependent osteoclastogenesis in patients affected by solid tumor. PLoS One. 2006;1:e124.

E16. Melchjorsen J, Risør MW, Søgaard OS, O'Loughlin KL, Chow S, Paludan SR, Ellermann-Eriksen S, Hedley DW, Minderman H, Østergaard L, Tolstrup M. Tenofovir selectively regulates production of inflammatory cytokines and shifts the IL-12/IL-10 balance in human primary cells. J Acquir Immune Defic Syndr. 2011;57:265-275.

E17. Onnis B, Fer N, Rapisarda A, Perez VS, Melillo G. Autocrine production of IL-11 mediates tumorigenicity in hypoxic cancer cells. J Clin Invest. 2013;123:1615-1629.

E18. Stellato C, Gubin MM, Magee JD, Fang X, Fan J, Tartar DM, Chen J, Dahm GM, Calaluce R, Mori F, Jackson GA, Casolaro V, Franklin CL, Atasoy U. Coordinate regulation of GATA-3 and Th2 cytokine gene expression by the RNA-binding protein HuR. J Immunol. 2011;187:441-449.

E19. Azimi N, Jacobson S, Leist T, Waldmann TA. Involvement of IL-15 in the pathogenesis of human T lymphotropic virus type I-associated myelopathy/tropical spastic paraparesis: implications for therapy with a monoclonal antibody directed to the IL-2/15R beta receptor. J Immunol. 1999;163:4064-4072.

E20. Rajput S, Volk-Draper LD, Ran S. TLR4 is a novel determinant of the response to paclitaxel in breast cancer. Mol Cancer Ther. 2013;12:1676-1687.

E21. Fujino S, Andoh A, Bamba S, Ogawa A, Hata K, Araki Y, Bamba T, Fujiyama Y. Increased expression of interleukin 17 in inflammatory bowel disease. Gut. 2003;52:65-70.

E22. Fort MM, Cheung J, Yen D, Li J, Zurawski SM, Lo S, Menon S, Clifford T, Hunte B, Lesley R, Muchamuel T, Hurst SD, Zurawski G, Leach MW, Gorman DM, Rennick DM. IL-25 induces IL-4, IL-5, and IL-13 and Th2-associated pathologies in vivo. Immunity. 2001;15:985-995.

E23. Wood IS, Wang B, Trayhurn P. IL-33, a recently identified interleukin-1 gene family member, is expressed in human adipocytes. Biochem Biophys Res Commun. 2009;384:105–109.

E24. Pinkas H, Fisch B, Rozansky G, Felz C, Kessler-Icekson G, Krissi H, Nitke S, Ao A, Abir R. Platelet-derived growth factors (PDGF-A and -B) and their receptors in human fetal and adult ovaries. Mol Hum Reprod. 2008;14:199-206.

E25. Dong Z, Nemeth JA, Cher ML, Palmer KC, Bright RC, Fridman R. Differential regulation of matrix metalloproteinase-9, tissue inhibitor of metalloproteinase-1 (TIMP-1) and TIMP-2 expression in co-cultures of prostate cancer and stromal cells. Int J Cancer. 2001;93:507-515.
